# Robot-assisted simple prostatectomy vs. laser enucleation of the prostate for large-volume benign prostatic hyperplasia (BPH, ≥80 mL): a systematic review and meta-analysis

**DOI:** 10.3389/fmed.2026.1804731

**Published:** 2026-05-05

**Authors:** Xiaoming Hao, Sicheng Wang, Fei Deng, Tongyi Li, Feng Chang

**Affiliations:** Department of Urology, Heping Hospital Affiliated to Changzhi Medical College, Changzhi, Shanxi, China

**Keywords:** benign prostatic hyperplasia, large-volume, laser enucleation, meta-analysis, robot-assisted simple prostatectomy

## Abstract

**Background:**

Robot-assisted simple prostatectomy (RASP) and laser enucleation of the prostate (LEP) are established surgical options for the treatment of large-volume benign prostatic hyperplasia (BPH); however, consensus regarding the superiority of one technique over the other has not been established. This study aimed to comprehensively compare the perioperative efficiency, safety, and functional outcomes of RASP and LEP.

**Methods:**

We conducted a comprehensive search of four databases (PubMed, Embase, Web of Science, and Scopus) to identify studies comparing RASP and LEP in large-volume BPH. Pooled and subgroup analyses were performed using Stata MP 18 and Review Manager 5.4.0.

**Results:**

Fifteen studies (2,231 patients, 763 RASP and 1,468 LEP) were included in the analysis. LEP was associated with shorter operative time and catheterization duration than RASP. Hospital stay was reduced with thulium fiber laser enucleation of the prostate (ThuLEP; mean difference [MD] 2.43, 95% confidence interval [CI] 1.52–3.34, *p* < 0.001), but not with holmium fiber laser enucleation of the prostate (HoLEP). Specimen weights were comparable overall (MD 3.61, 95% CI −11.29–18.51, *p* = 0.60), although ThuLEP yielded smaller specimens (MD 28.44, 95% CI 12.17–44.71, *p* = 0.01). Low- and high-grade Clavien–Dindo complications were comparable between groups, while RASP was associated with incidence of lower urinary incontinence (OR 0.48, 95% CI 0.27–0.85, *p* = 0.01) and superior improvements in International Prostate Symptom Score (MD −1.33, 95% CI −2.41–−0.26, *p* = 0.015) and post-void residual (MD −3.95, 95% CI −7.28–−0.61, *p* = 0.020), with International Prostate Symptom Score benefit observed primarily at long-term follow-up (≥12 months, MD −1.45, 95% CI −2.24–−0.67, *p* = 0.012). Maximum urinary flow rate and quality of life were comparable across groups.

**Conclusion:**

Both RASP and LEP may be safe and effective surgical options for large-volume BPH. LEP may be associated with faster perioperative recovery, whereas RASP may reduce the incidence of UI and potentially improve long-term symptom control. Given the observational design of included studies, heterogeneity across cohorts, and limitations of retrospective evidence, these findings should be interpreted with caution, and tailored surgical strategies are recommended.

## Introduction

Benign prostatic hyperplasia (BPH) is among the most prevalent urological disorders in aging males, with an incidence that increases progressively with age. Specifically, the prevalence reaches 40–50% in men aged 51–60 years and exceeds 80% in those aged 80 years and older (1, 2). Large-volume BPH (≥80 mL) poses distinct surgical challenges owing to the increased tissue burden, which is associated with increased perioperative complications, longer operative time, and more technically challenging dissection (3). Therefore, identifying the optimal surgical approach for large-volume BPH remains an important clinical priority.

Minimally invasive surgery has broadened the therapeutic options for BPH, with robot-assisted simple prostatectomy (RASP) and laser enucleation of the prostate (LEP)—primarily holmium laser enucleation of the prostate (HoLEP) and thulium laser enucleation of the prostate (ThuLEP)—being the most widely adopted techniques (4, 5). RASP combines the benefits of minimally invasive surgery with the thoroughness of open surgery (6). LEP uses specific laser energy to enable precise dissection and hemostasis, serving as an important alternative to RASP (4). Notably, HoLEP and ThuLEP exhibit key differences in their laser characteristics and tissue interactions (7). HoLEP uses a 2,140 nm holmium laser, which enables efficient tissue ablation and reliable hemostasis through high-power vaporization and sharp incision. In contrast, ThuLEP utilizes a 1940 nm thulium laser; its wavelength closely matches the water absorption peak in prostatic tissue, allowing for more precise dissection, enhanced hemostatic control, and a narrower zone of thermal injury. These inherent differences may explain the divergent perioperative and functional outcomes observed in our subgroup analysis when comparing each technique to RASP.

Despite their widespread adoption, considerable controversy remains regarding the relative efficacy and safety of RASP and LEP. Previous studies have reported inconsistent findings, with most focusing on a single laser modality (HoLEP or ThuLEP) rather than evaluating them collectively as a unified LEP approach (8, 9). To the best of our knowledge, only a few meta-analyses have compared RASP with combined LEP (HoLEP and ThuLEP) specifically for large-volume BPH (10). However, these analyses were constrained by the small number of included studies, potentially limiting their statistical power and generalizability. Consequently, reliable and comprehensive evidence to support this comparison remains lacking.

Therefore, to address this gap, this meta-analysis aimed to compare RASP with LEP by including additional relevant studies to enhance the robustness of the results.

## Methods

This systematic review and meta-analysis was conducted in accordance with the Preferred Reporting Items for Systematic Reviews and Meta-Analyses guidelines (11) and was registered with PROSPERO (CRD420251275935). Two minor protocol amendments were implemented: (1) the intervention was specified as robot-assisted simple prostatectomy (RASP) to match the included studies, and (2) the search period was extended to database inception to enhance statistical power. However, the primary research question remained unchanged. Ethical approval was waived because this study used only publicly available anonymized data from previously published studies.

### Search strategy

We conducted a comprehensive literature search of Web of Science, Embase, PubMed, and Scopus from their inception to December 31, 2025, with no language restrictions. The search terms were as follows: “robot-assisted simple prostatectomy,” “RASP,” “holmium laser enucleation of the prostate”, “HoLEP,” “thulium laser enucleation of the prostate,” “ThuLEP,” “benign prostatic hyperplasia,” “BPH,” “benign prostatic enlargement,” and “BPE.” Two authors independently reviewed the literature and any discrepancies arising during the screening process were resolved through discussion with a third reviewer.

### Identification of eligible studies

Studies were included if they met the following inclusion criteria: (1) randomized controlled trials (RCTs) or non-randomized controlled studies comparing RASP with HoLEP or ThuLEP; (2) adult male patients with BPH and a prostate volume ≥80 mL or weight ≥80 g (volume confirmed by ultrasound/MRI and weight of surgical specimen); (3) published or preprinted before December 31, 2025; (4) reported sufficient data for at least one predefined outcome (perioperative outcomes, functional parameters, or complications). Case reports, reviews, meta-analyses, editorials, commentaries, and laboratory studies were also excluded.

### Data extraction

Relevant data were independently extracted from each included study by two reviewers, with discrepancies resolved through discussion with a third independent reviewer. Extracted data included publication year, first author, country of origin, study period, study design, number of patients, follow-up duration, patient age, prostate volume/mass, operative time, catheterization duration, length of hospital stay, specimen weights, Clavien–Dindo complications, International Prostate Symptom Score (IPSS), quality of life (QoL) score, maximum urinary flow rate (Q_max_), and post-void residual (PVR). Where necessary, raw data from eligible primary studies were converted to mean values and standard deviations (SD) using established computational formulae (12). If possible, subgroup analyses were performed to compare urinary incontinence rates and Clavien–Dindo classification (CDC) grades between the RASP and LEP groups.

### Quality assessment

Methodological quality was evaluated using the Newcastle (Ottawa) scale, with assessments centered on three core dimensions: study group selection, intergroup comparability, and exposure/outcome evaluation (13). In this meta-analysis, studies scoring ≥7 were regarded as high-quality. Two reviewers independently appraised the methodological quality of each study and any discrepancies were resolved through consultation with a third reviewer.

### Statistical analysis

Data extracted from eligible studies were analyzed using StataMP 18 (StataCorp, College Station, TX, United States) and Review Manager (RevMan) version 5.4.0.^1^ Treatment effects were assessed by comparing baseline and endpoint values. Continuous variables were reported as mean differences (MDs) or standardized mean differences (SMDs) with 95% confidence intervals (CIs), while dichotomous variables were reported as odds ratios (ORs) with 95% CIs. Between-study heterogeneity was evaluated using the chi-square test and I^2^ statistic. A random-effects model was applied for pooled analyses. Sensitivity analyses were conducted by sequentially excluding studies that contributed to heterogeneity and publication bias was assessed using funnel plots and Begg’s tests.

## Results

### Search results and study characteristics

**Figure 1:**
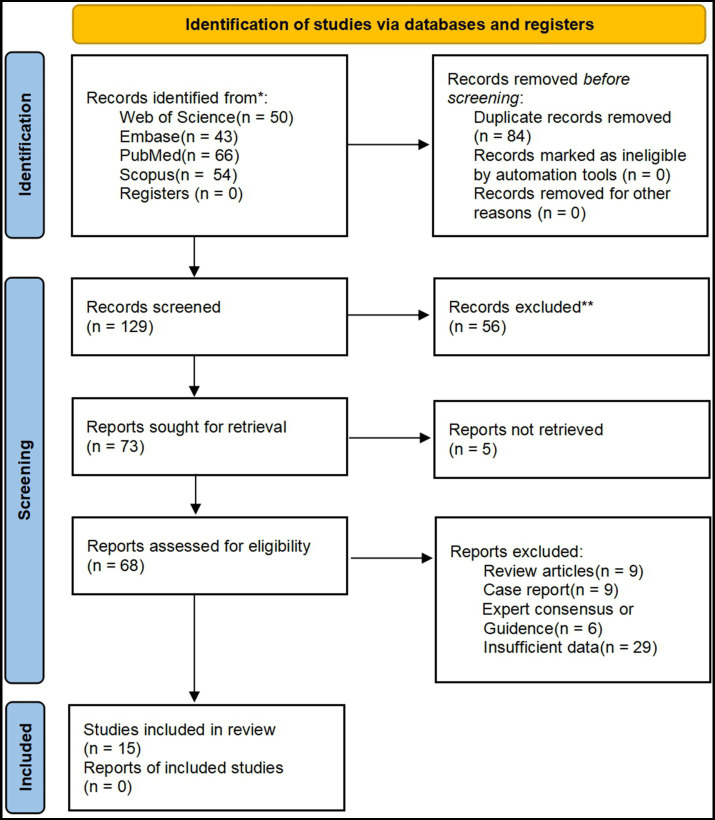
PRISMA flow diagram of literature screening and eligibility assessment.

Figure 1 depicts the Preferred Reporting Items for Systematic Reviews and Meta-Analyses flow diagram for literature identification and eligibility assessment. A total of 213 records were retrieved from the four databases, with no prescreening exclusions. After title and abstract screening, 56 records were excluded owing to obvious irrelevance. Of the remaining 73 studies, five full texts were unavailable, leaving 68 articles for eligibility assessment. After full-text review, 53 articles were excluded, resulting in 15 studies being included in this systematic review and meta-analysis. No supplementary studies were identified.

### Study selection and characteristics of included studies

Fifteen eligible studies encompassing 2,231 patients were included in this meta-analysis, including 14 retrospective studies and one prospective observational study (14–28). Among these, 10 studies (*n* = 1,563) compared RASP with HoLEP (14–23), while five studies (*n* = 668) assessed RASP versus ThuLEP (24–28). Detailed study characteristics (Table 1) and baseline clinical characteristics of the enrolled patients (Table 2) are summarized in Supplementary material 1. Quality assessment demonstrated that all studies met the criteria for high methodological quality, and specific scoring details for each study are provided in Supplementary material 2.

**Table 1:** Study characteristics.

Study	Country	Research period(y)	Study design	Intervention	Patients (n)	Age (y)	Min volume/mass (ml/g)	IPSS	Quality score
Chou et al. (14)	USA	2018–2022	Retrospective	RASP	80	76.0 ± 5.9	≥80 g	26.5 ± 5.3	8
				HoLEP	80	74.0 ± 9.0		27.0 ± 5.2	
Hartung et al. (15)	Germany	2015–2021	Retrospective	RASP	39	70.6 ± 7.9	≥80 ml	21.4 ± 6.4	8
				HoLEP	38	70.7 ± 8.6		18.5 ± 7.5	
Silvia et al. (16)	Spain	2007–2023	Retrospective	RASP	50	72.4 ± 7.8	≥150 ml	21.9 ± 4.9	7
				HoLEP	95	72.4 ± 8.9		21.7 ± 4.9	
Bove et al. (17)	Italy	2011–2021	Retrospective	RASP	43	72.0 ± 8.1	≥80 g	20.0 ± 3.0	8
				HoLEP	54	70.0 ± 5.9		20.0 ± 5.9	
Palacios et al. (18)	USA	2019–2022	Retrospective	RASP	50	70.0 ± 5.9	≥80 ml	23.0 ± 9.6	8
				HoLEP	90	72.0 ± 5.2		18.0 ± 9.6	
Vander et al. (19)	Belgium	2009–2020	Retrospective	RASP	31	73.0 ± 9.3	≥200 mL	18.0 ± 9.6	8
				HoLEP	22	77.5 ± 7.4		15.0 ± 5.2	
Kim et al. (20)	Korea	2018–2021	Retrospective	RASP	33	68.1 ± 10.1	≥80 ml	NA	7
				HoLEP	26	70.8 ± 7.6			
Fuschi et al. (21)	Italy	2016–2018	Prospective	RASP/LSP	68	66.4 ± 7.5	≥120 mL	23.9 ± 1.9	8
				HoLEP	42	68.2 ± 6.1		24.2 ± 3.0	
Umari et al. (22)	Italy	2008–2015	Retrospective	RASP	81	69.0 ± 7.4	≥100 mL	25.0 ± 5.9	8
				HoLEP	45	74.0 ± 8.9		21.0 ± 6.7	
Zhang et al. (23)	USA	2008–2015	Retrospective	RASP	32	71.0 ± 8.0	≥80 g	24.0 ± 4.0	8
				HoLEP	600	71.0 ± 8.0		20.0 ± 7.0	
Audige et al. (24)	France	2020–2023	Retrospective	RASP	106	70.5 ± 5.9	≥80 ml	20.9 ± 6.2	8
				ThuLEP	128	71.9 ± 8.5		17.6 ± 7.6	
Perri et al. (25)	Italy	NA	Retrospective	RASP	100	70.2 ± 8.3	≥150 ml	24.3 ± 5.8	8
				ThuLEP	100	74.5 ± 7.6		22.5 ± 7.6	
Hartung et al. (26)	Germany	2015–2021	Retrospective	RASP	38	70.0 ± 8.0	≥80 ml	NA	7
				ThuLEP	38	70.0 ± 6.0			
Susan et al. (27)	USA	2017–2021	Retrospective	RASP	33	68.0 ± 5.9	≥80 g	26.5 ± 6.7	7
				ThuLEP	69	68.0 ± 8.1		22.0 ± 12.6	
Hou et al. (28)	China	2014–2020	Retrospective	RASP	15	66.4 ± 6.4	≥80 g	26.3 ± 5.1	7
				ThuLEP	41	71.9 ± 8.5		25.1 ± 5.5	

RASP, robot-assisted simple prostatectomy; HoLEP, holmium laser enucleation of the prostate; ThuLEP, thulium laser enucleation of the prostate; IPSS, international prostate symptom score; NA, not available.

**Table 2:** Baseline characteristics of patients.

Study	Intervention	BMI (kg/m^2^)	PSA (ng/mL)	PV (mL)	QoL	Qmax (mL/s)	Indwelling catheter(n)
Chou et al. (14)	RASP	28.5 ± 5.2	5.2 ± 2.5	NA	NA	NA	7
	HoLEP	26.5 ± 5.3	5.5 ± 3.7				11
Hartung et al. (15)	RASP	29.0 ± 5.9	10.1 ± 6.1	186.7 ± 55.7	NA	NA	27
	HoLEP	30.2 ± 6.2	12.3 ± 10.8	184.8 ± 54.9			13
Silvia et al. (16)	RASP	NA	7.7 ± 4.2	203.4 ± 98.1	NA	8.1 ± 3.5	10
	HoLEP		8.73 ± 8	187.7 ± 45.9		10.9 ± 5.8	40
Bove et al. (17)	RASP	25.0 ± 4.4	4.9 ± 4.5	105.0 ± 25.9	3.0 ± 0.7	7.0 ± 4.4	NA
	HoLEP	26.0 ± 3.0	5.1 ± 3.3	102.0 ± 20.7	3.0 ± 0.7	7.0 ± 2.2	
Palacios et al. (18)	RASP	29.0 ± 4.4	9.0 ± 5.9	169.0 ± 71.1	5.0 ± 1.5	6.0 ± 1.5	26
	HoLEP	26.0 ± 4.4	6.0 ± 4.4	129.0 ± 37.0	4.0 ± 1.5	6.0 ± 3.7	50
Vander et al. (19)	RASP	27.6 ± 3.9	10.4 ± 9.0	225.0 ± 29.6	4.0 ± 1.5	10.0 ± 5.2	8
	HoLEP	26.1 ± 4.0	7.5 ± 3.0	204.5 ± 14.8	4.0 ± 0.7	8.2 ± 0.9	3
Kim et al. (20)	RASP	NA	NA	97.9 ± 37.9	NA	NA	NA
	HoLEP			84.1 ± 31.3	NA	NA	NA
Fuschi et al. (21)	RASP/LSP	14.0 ± 20.5	5.5 ± 3.0	146.9 ± 30.7	3.9 ± 0.9	7.2 ± 1.3	16
	HoLEP	23.5 ± 3.3	5.6 ± 3.3	142.2 ± 30.1	3.9 ± 0.8	7.1 ± 1.9	11
Umari et al. (22)	RASP	27.0 ± 5.2	7.1 ± 6.2	130.0 ± 27.4	NA	8.0 ± 4.4	9
	HoLEP	26.0 ± 3.7	8.6 ± 8.4	130.0 ± 58.5		9.0 ± 5.2	4
Zhang et al. (23)	RASP	NA	NA	NA	NA	NA	11
	HoLEP						140
Audige et al. (24)	RASP	26.5 ± 2.9	10.9 ± 7.2	135.2 ± 39.7	4.4 ± 0.7	8.9 ± 3.0	30
	ThuLEP	26.6 ± 4.0	7.3 ± 7.9	106.4 ± 26.8	4.1 ± 1.5	10.4 ± 7.2	29
Perri et al. (25)	RASP	NA	4.9 ± 2.3	186.3 ± 12.3	NA	7.2 ± 3.3	14
	ThuLEP		4.2 ± 1.7	178.6 ± 11.4		9.0 ± 2.8	12
Hartung et al. (26)	RASP	29.0 ± 6.0	NA	184.0 ± 51.0	NA	NA	NA
	ThuLEP	29.0 ± 4.0		179.0 ± 44.0			
Susan et al. (27)	RASP	28.2 ± 5.0	NA	120.0 ± 204.4	NA	NA	14
	ThuLEP	28.1 ± 4.0		180.0 ± 334.8			32
Hou et al. (28)	RASP	NA	10.4 ± 5.4	116.4 ± 17.9	5.3 ± 0.6	5.4 ± 1.8	4
	ThuLEP		8.7 ± 7.5	89.8 ± 7.8	5.1 ± 0.7	6.7 ± 4.1	17

BMI, body mass index; PSA, prostate-specific antigen; QoL, quality of life score; Qmax, maximum urinary flow rate; PVR, postvoid residual volume; PV, prostate volume; RASP, robot-assisted simple prostatectomy; HoLEP, holmium laser enucleation of the prostate; ThuLEP, thulium laser enucleation of the prostate; NA, not available.

### Perioperative outcomes

Pooled analysis demonstrated that LEP was associated with significantly shorter operative time compared with RASP (MD 31.56, 95% CI 10.97–52.14, *p* = 0.003). Similarly, both catheterization duration (MD 3.51, 95% CI 2.35–4.66, *p* < 0.001) and hospital stay (MD 1.61, 95% CI 0.57–2.64, *p* = 0.002) were significantly reduced in the laser group. However, no significant difference in resected specimen weight was observed between the two groups.

Subgroup analyses confirmed that both HoLEP and ThuLEP were associated with shorter operative times and reduced catheterization durations compared with RASP. Hospital stays were significantly shorter in the ThuLEP subgroup (MD 2.43, 95% CI 1.91–2.95, *p* < 0.001). Notably, ThuLEP yielded smaller resected specimen weights than RASP (MD 28.44, 95% CI 16.62–40.26, *p* = 0.001; Table 3).

**Table 3:** Subgroup analysis of perioperative outcomes and complications comparing RASP and LEP.

Outcome	Subgroup type	Subgroup category	Patients (RASP/Laser)	MD/OR (95%CI)	*p*	Heterogeneity	
						I^2^	*p*
LOS	All		649/814	1.61 (0.57, 2.64)	0.002	99%	<0.0001
	Laser	RASP vs. HoLEP	390/507	1.21 (−0.08, 2.50)	0.07	99%	<0.0001
		RASP vs. ThuLEP	259/307	2.43 (1.91, 2.95)	<0.0001	58%	0.007
SW	All		419/1065	3.61 (−10.02, 17.24)	0.60	88%	<0.0001
	Laser	RASP vs. HoLEP	348/958	−1.94 (−16.30, 12.42)	0.79	87%	<0.0001
		RASP vs. ThuLEP	71/107	28.44 (16.62, 40.26)	0.001	0%	0.36
ICT	All		537/1212	3.51 (2.35, 4.66)	<0.0001	97%	<0.0001
	Laser	RASP vs. HoLEP	260/877	3.52 (1.87, 5.17)	<0.0001	98%	<0.0001
		RASP vs. ThuLEP	277/335	3.40 (2.98, 3.83)	<0.0001	0%	0.44
OT	All		763/1468	31.56 (10.97, 52.14)	0.003	98%	<0.0001
	Laser	RASP vs. HoLEP	471/1092	28.19 (0.95, 55.44)	0.04	97%	<0.0001
		RASP vs. ThuLEP	292/376	38.50 (2.33, 74.68)	0.04	98%	<0.0001
Overall complications*	All		643/1239	1.25 (0.71, 2.20)	0.44	0%	0.82
	Laser	RASP vs. HoLEP	464/1038	1.85 (0.91, 3.77)	0.09	0%	0.95
		RASP vs. ThuLEP	179/201	0.54 (0.19, 1.55)	0.25	0%	0.77
Transfusions*	All		672/788	0.94 (0.55, 1.61)	0.51	27%	0.20
	Laser	RASP vs. HoLEP	410/481	0.87 (0.44, 1.72)	0.70	0%	0.56
		RASP vs. ThuLEP	262/307	0.74 (0.27, 2.05)	0.56	72%	0.03

ICT, indwelling catheter time; LOS, length of hospital stay; SW, specimen weight; OT, operative time; CDC, Clavien–Dindo classification; UI, urinary incontinence; RASP, robot-assisted simple prostatectomy; HoLEP, holmium laser enucleation of the prostate; ThuLEP, thulium laser enucleation of the prostate; MD (95%CI), mean difference (95% confidence interval); OR, odds ratio. *Indicates the use of OR as an effect statistic.

Notably, substantial heterogeneity (I^2^ > 90%) was observed for those outcomes. To explore potential sources of this variation, multivariate meta-regression was performed using mean prostate volume, publication year, study design, and total sample size as covariates. No variable was identified as a significant moderator of heterogeneity (*p* > 0.05).

### Complications and urinary incontinence

**Figure 2:**
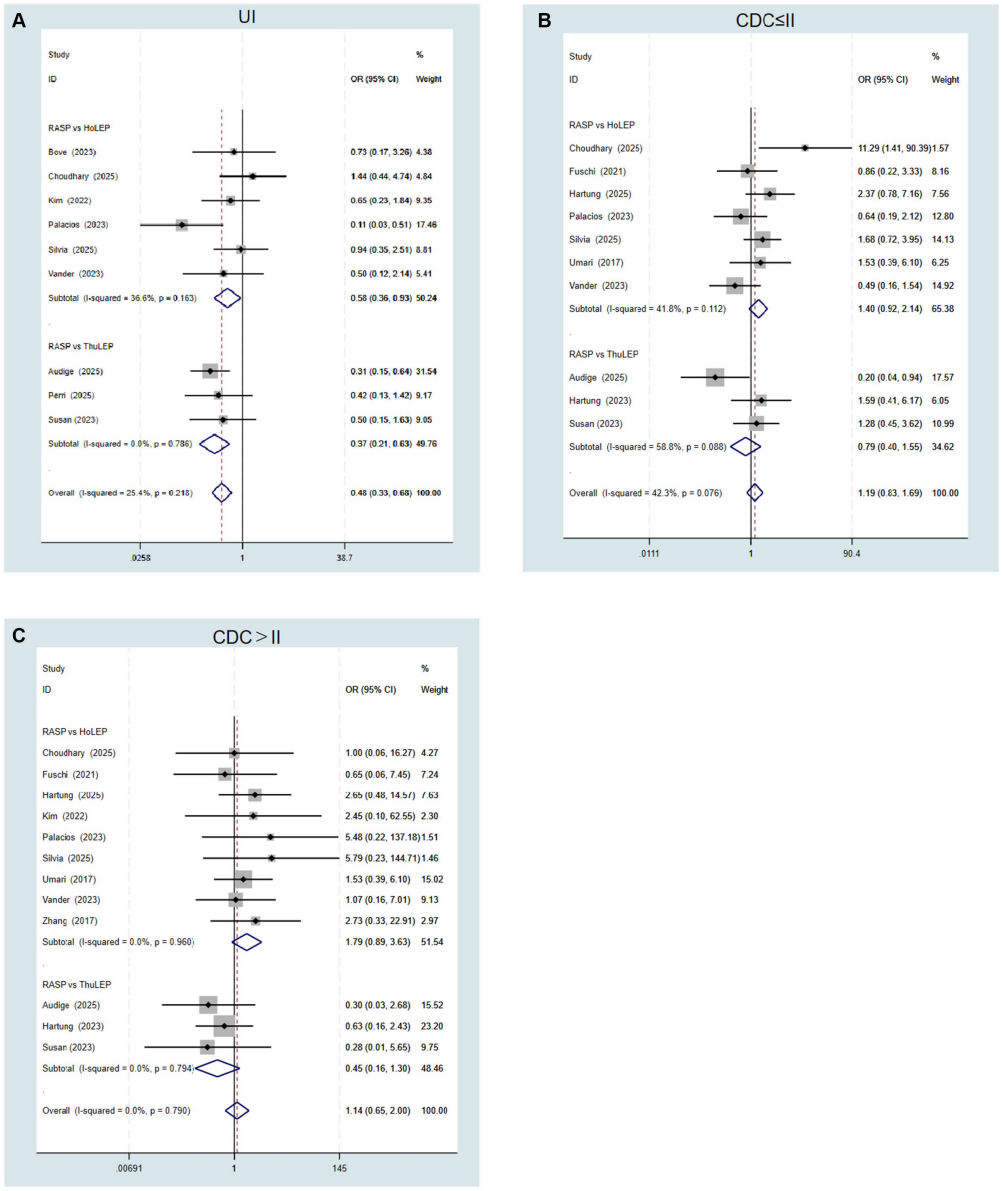
Forest plots. **(A)** Urinary incontinence (UI); **(B)** Low-grade Clavien–Dindo classification of complications (CDC ≤ II); and **(C)** High-grade Clavien–Dindo classification of complications (CDC > II).

Pooled analysis demonstrated no significant differences between the RASP and LEP groups in overall or transfusion complications (Table 3). Nine studies (14, 16–20, 24, 25, 27) reported postoperative urinary incontinence (UI). Compared with LEP, RASP was associated with a significantly lower incidence of pooled overall UI (OR 0.48, 95% CI 0.33–0.68, *p* = 0.01; Figure 2A).

Subgroup analyses confirmed comparable safety profiles across laser modalities. The rates of both low-grade (CDC ≤ II) and high-grade (CDC > II) complications, as well as transfusion requirements, were similar between groups (Figures 2B,C). However, RASP consistently surpassed both HoLEP (OR 0.58, 95% CI 0.36–0.93, *p* = 0.02, I^2^ = 36%) and ThuLEP (OR 0.37, 95% CI 0.21–0.63, *p* = 0.01, I^2^ = 0%) in terms of mitigating pooled overall UI (Figure 2A).

### Efficacy outcomes

**Figure 3:**
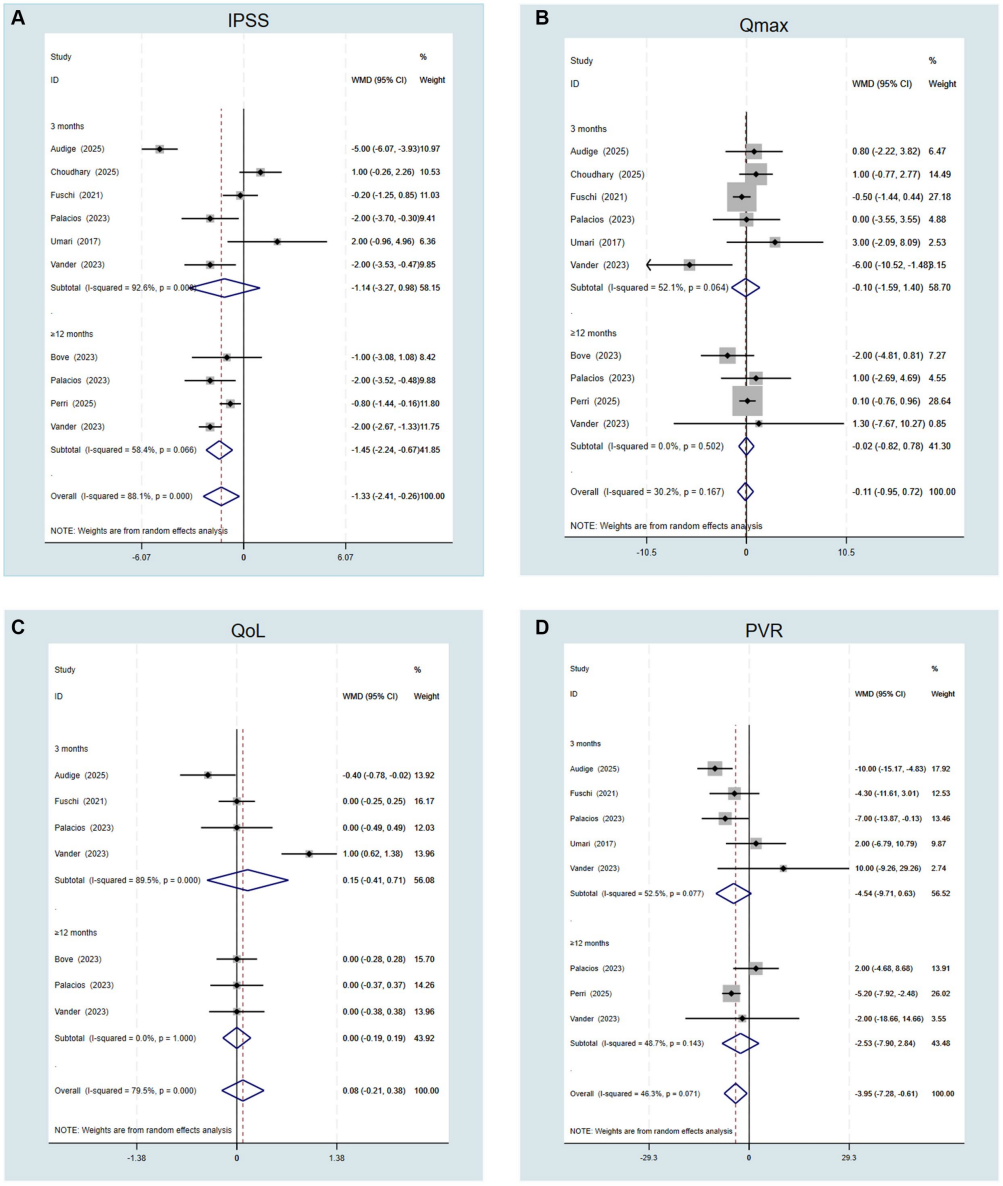
Forest plots of efficacy outcomes. **(A)** International Prostate Symptom Score (IPSS); **(B)** Maximum urinary flow rate (Q_max_); **(C)** Quality of life (QoL); and **(D)** Post-void residual (PVR).

Six short-term (≤3 months) studies (14, 18, 19, 21, 22, 24)and four long-term (≥12 months) studies (17–19, 25) were identified and included for assessment of IPSS and Q_max_. Pooled analysis suggested that RASP was associated with superior functional outcomes compared with LEP, including lower IPSS (MD −1.33, 95% CI −2.41 – −0.26, *p* = 0.015) and PVR (MD −3.95, 95% CI −7.28 – −0.61, *p* = 0.020; Figures 3A,D). However, no significant differences were observed in Q_max_ or QoL between the two groups (Figures 3B,C).

Subgroup analysis by follow-up duration demonstrated that the superiority of RASP compared with LEP in IPSS was maintained at long-term follow-up (≥12 months; MD −1.45, 95% CI −2.24 – −0.67, *p* = 0.001; Figure 3A). In contrast, Q_max_, QoL, and PVR remained comparable between the groups across both short-term (≤3 months) and long-term (≥12 months) follow-up periods (Figures 3B–D). High between-study heterogeneity (I^2^ > 90%) was also observed for IPSS. As reported above, meta-regression did not identify significant sources of this heterogeneity (*p* > 0.05).

### Sensitivity analysis

A leave-one-out sensitivity analysis was performed using StataMP 18 software and the robustness of our findings was confirmed. Sequential exclusion of each individual study did not materially alter the pooled effect estimates or heterogeneity, confirming the stability of this meta-analysis (Supplementary material 3). Beyond leave-one-out analysis, we further assessed result robustness by repeating our primary analyses after excluding studies at high risk of bias (based on Cochrane risk-of-bias assessment) and those with small sample sizes (fewer than 100). Pooled estimates for these key outcomes remained stable across all analyses.

### Subgroup analysis

Subgroup analyses were conducted according to laser type, follow-up duration, and high- and low-grade CDC complications. The results are presented alongside the overall pooled analysis. An additional subgroup analysis of UI subtypes was planned but was not feasible owing to insufficient data.

### Publication bias

**Figure 4:**
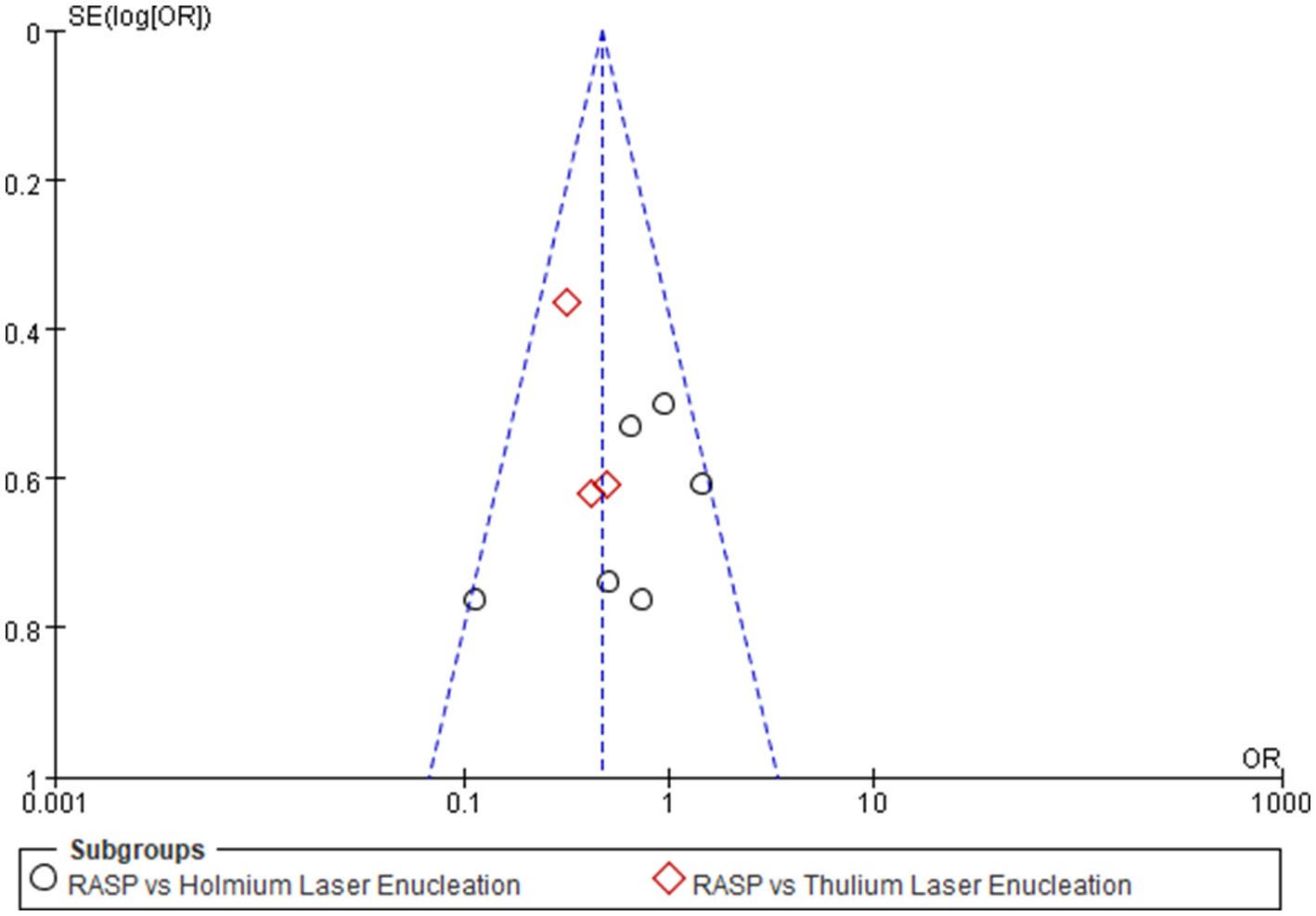
Funnel plot for assessment of publication bias.

Publication bias was assessed using UI as the primary outcome measure. Visual inspection of the funnel plot asymmetry (Figure 4), Begg’s test (*p* = 0.12) and Egger’s test (*p* = 0.26) revealed no significant evidence of publication bias.

## Discussion

Large-volume BPH is a common urological disorder that frequently requires surgical intervention after failure of conservative management. LEP (including HoLEP and ThuLEP) and RASP are effective first-line therapeutic modalities for large-volume BPH that are associated with minimal perioperative complications and serve as alternatives to traditional open surgery (1). However, the optimal strategy (RASP or LEP) remains uncertain owing to inconsistent clinical findings (9, 17, 28). It should be noted that only 2 of the included studies used single-port RASP; all other RASP procedures were conventional multi-port.

Shuai et al. (10) previously conducted a meta-analysis comparing RASP and LEP (HoLEP and ThuLEP) for large-volume BPH and reported that LEP was associated with a significantly shorter operative time (*p* = 0.04), length of hospital stay (*p* < 0.01), and catheterization duration (*p* < 0.01) compared with RASP. Specimen weights and functional outcomes, including IPSS, Qmax, and PVR, were comparable between groups. LEP was also associated with a lower transfusion rate (*p* = 0.006) and a lower rate of major complications (defined as CDC grade >II; *p* = 0.049). However, this analysis only included only six studies with 1,235 patients and assessed functional outcomes at a relatively short follow-up duration of 3 months. Currently, comprehensive, large-sample, and long-term follow-up studies comparing these two surgical modalities are lacking. To ensure methodological comparability with RASP, this meta-analysis focused exclusively on HoLEP and ThuLEP, the predominant anatomical enucleation techniques within the LEP category.

The comprehensive comparison between RASP and LEP was conducted in the current meta-analysis using data from 15 studies involving 2, 231 patients. Perioperative efficacy, safety profiles, and short- and long-term functional outcomes were analyzed. Furthermore, subgroup analyses were conducted according to laser type to facilitate direct comparison with RASP and to provide high-quality evidence to guide clinical decision-making. Our findings confirmed that LEP was associated with shorter operative time and catheterization duration, while ThuLEP was associated with a shorter hospital stay and a smaller specimen weight. Overall complication rates (low- and high-grade) were comparable between groups, indicating similar safety profiles. Regarding functional outcomes, RASP achieved superior IPSS and PVR results, particularly at long-term follow-up (≥12 months). Whereas Q_max_ and QoL were comparable across all groups regardless of the follow-up duration. Notably, RASP showed a significantly lower incidence of UI than LEP (*p* = 0.01), and this advantage was consistently demonstrated in both the HoLEP and ThuLEP subgroups. However, this conclusion should be further validated through high-quality RCTs and more specific subgroup analyses.

Perioperative outcomes are particularly important in elderly patients with large-volume BPH and concurrent comorbidities. In our study, the perioperative advantages of LEP over RASP were confirmed, with significantly shorter operative times (*p* = 0.003), shorter catheterization durations (*p* < 0.001), and shorter hospital stays (*p* = 0.002). Although a previous small-sample study (10) reported even greater advantages of LEP, these differences may reflect the small sample size and relatively basic techniques. In the past 2 years, technical advancements and the adoption of standardized protocols for both modalities may have contributed to a narrowing of the gap between approaches.

Further subgroup analyses showed that both HoLEP and ThuLEP had shorter operative times and catheterization durations than RASP, while ThuLEP significantly shortened the hospital stay. The rapid perioperative recovery advantage exhibited by LEP is consistent with the results of many previous studies (29, 30). Notably, the ThuLEP-based subgroup analysis markedly reduced heterogeneity in catheterization duration, hospital stay, and specimen weights, suggesting that the inter-study variability in these outcomes was largely attributed to differences in LEP subtypes. This also improved the consistency and reliability of the results. In contrast, the subgroup analysis failed to reduce heterogeneity in operative time, indicating that this variability was unrelated to surgical subtype, but may be associated with surgeon expertise, prostate volume, or surgical equipment. No significant difference in specimen weight was observed between LEP and RASP, consistent with the findings of previous studies (20, 21, 26) and a similar meta-analysis (10), demonstrating that both surgical approaches achieve effective resection of the prostatic tissue. However, the prior analysis included only one ThuLEP study and five HoLEP studies. The limited ThuLEP data and the absence of subgroup analyses likely masked the observed modality-specific specimen weight differences.

In our study, subgroup analysis demonstrated that RASP yielded significantly greater specimen weights than ThuLEP, consistent with other reports (28). Potential reasons for this may be precise resection of the gland, possible laser-induced partial tissue vaporization, and variations in operator proficiency. Conflicting evidence remains with some studies suggesting more complete resection with HoLEP (19, 22) and others with RASP (18), underscoring the influence of laser parameters, surgeon experience, and robotic equipment on resection outcomes.

Notably, operative time, catheter duration and hospital stay demonstrated considerable heterogeneity across the analysis. Such variability may reflect differences in surgical techniques, surgeon experience, perioperative management, baseline patient characteristics, and outcome definitions across study populations and centers. Although meta-regression was performed to explore potential sources of heterogeneity, no significant moderators were identified. This may be partly attributed to the relatively small number of included studies and inherent differences across study populations and surgical practices. In addition, both LEP and RASP are highly technique-dependent procedures. Their clinical outcomes are closely tied to surgeon proficiency, and the learning curve may exert a substantial impact on perioperative and functional results, as supported by previous studies (31, 32). Variability in surgical expertise across centers could contribute to heterogeneity and affect the robustness of pooled estimates. Thus, these potential influences should be taken into account when interpreting the present findings.

The present study found no significant differences between RASP and LEP in overall complications (*p* = 0.44) and blood transfusion (*p* = 0.51). Subgroup analysis further verified that complication rates were comparable between the two surgical approaches across laser subtypes. Stratification by CDC demonstrated no significant differences in either low- (CDC ≤ II) or high-grade (CDC > II) complications. These findings align with the results of multiple studies (8, 25, 26), supporting the safety of both modalities in the management of large-volume BPH.

In contrast, several meta-analyses reported lower rates of high-grade complications and blood transfusions with LEP (10, 30), potentially reflecting the inherent technical properties of laser systems. Optimized laser parameters may enhance hemostatic efficacy and precision of tissue ablation (33), while increasing surgeon proficiency with laser energy modulation and control may further mitigate complications. In addition, RASP was associated with a significantly lower overall incidence of postoperative UI than LEP, regardless of laser type. A previous small-sample meta-analysis did not compare postoperative UI between LEP and RASP, and our study helps to address this gap. Consistent with our findings, several studies have also shown superior postoperative UI with RASP (18, 23, 30). This superior outcome is likely attributable to its more conservative resection strategy, reduced dependence on equipment, and improved preservation of urethral function. In contrast, LEP may increase the risk of postoperative UI owing to thermal tissue injury (34), extensive dissection (29), and greater reliance on technical precision. Owing to inherent data limitations, we only assessed the overall incidence of UI, without distinguishing between its distinct clinical subtypes. Perri et al. (25) further distinguished between types of UI, noting that higher rates of temporary urgency (*p* = 0.03) and urge incontinence (*p* = 0.04) were observed after ThuLEP, with no significant differences in stress incontinence between ThuLEP and RASP. This finding suggests that RASP may provide superior short-term urinary control. However, evidence regarding long-term UI in RASP and LEP remains limited and warrants further investigation. Notably, substantial heterogeneity was observed in the definition of UI across included studies. Variations existed in postoperative follow-up time points, classification of transient versus persistent UI, and pad-use thresholds for continence. Given the limited number of studies and highly inconsistent reporting, subgroup analyses stratified by UI definition were not feasible. To test the robustness of our findings, we performed a leave-one-out sensitivity analysis. The results confirmed that the pooled effect estimate remained stable. Due to this definitional variability, our results should be interpreted with appropriate caution. Future prospective studies with standardized UI assessment criteria are warranted to further validate these findings.

We compared postoperative functional outcomes between RASP and LEP across four core indicators and performed subgroup analyses stratified by follow-up duration to explore temporal variations in surgical efficacy. In the pooled analysis, RASP demonstrate statistically superior IPSS (*p* = 0.01) and PVR (*p* = 0.02) compared with LEP. No significant differences in IPSS were observed between the two groups at the short-term follow-up (≤3 months), with both procedures showing comparable short-term improvements in PVR, Q_max_, and QoL. These results are consistent with previous evidence. Shuai et al. (10) compared IPSS, Q_max_, and PVR between RASP and LEP at the 3-month follow-up, reporting no significant differences. Another meta-analysis comparing IPSS, Q_max_, and PVR at the 3-month and 6-month follow-ups revealed a higher PVR at the 6-month follow-up with RASP (6). Moreover, short-term outcomes for RASP and endoscopic enucleation of the prostate (EEP) were comparable in terms of IPSS and Q_max_; however, EEP in that study was performed using surgical rather than laser-based approaches and long-term stratified analyses were lacking. Interestingly, our long-term analysis (≥12 months) showed that RASP provided superior IPSS improvement compared with LEP (*p* = 0.01), whereas PVR, Q_max_, and QoL remained comparable. These findings may suggest a potential long-term advantage of RASP over LEP in IPSS improvement, although this observation is based on a limited number of studies with follow-up ≥12 months and should therefore be interpreted with caution. Although resection adequacy was comparable between groups, long-term IPSS outcomes differed because functional recovery depends not only on tissue resection but also on perioperative tissue injury, inflammation and surgical trauma. This time-specific advantage reflects more stable, long-term preservation of urethral function after RASP, whereas laser-induced thermal tissue injury may delay functional recovery. In addition, repeated transurethral instrument manipulation during LEP may cause mechanical trauma to the urethral mucosa, further contributing to delayed functional recovery and relatively inferior long-term IPSS improvement.

Although previous meta-regression did not identify prostate volume as a significant source of heterogeneity, this may be attributable to the limited number of studies and available data. In addition, although we set a minimum prostate volume threshold of 80 mL for study inclusion, none of the included studies reported a predefined upper limit. This led to a wide and unstratified range of prostate volumes across the included studies. Prostate volume is closely associated with surgical complexity, intraoperative performance, and postoperative recovery. Such unstratified variability may contribute to increased heterogeneity and compromise the stability of the pooled results. As highlighted in previous clinical research, prostate volume may independently influence surgical outcomes, which underscores this important limitation of our meta-analysis.

This study had several limitations. First, subgroup stratification by prostate volume was not feasible owing to the absence of a defined upper threshold across included studies. Second, significant heterogeneity was noted across several outcomes, likely driven by variations in surgeon experience, institutional practice patterns, surgical techniques, and perioperative protocols. Third, data on postoperative pain, urethral stricture, and sexual function were scarce, limiting further pooled analysis. Fourth, nearly all studies were retrospective in design, with few prospective data available. Such a predominantly observational design carries inherent risks of selection bias and confounding. Importantly, outcomes may also be affected by inter-surgeon expertise and center-level variability. These factors may limit the generalizability of the findings and should be considered when interpreting the results.

## Conclusion

The present study suggests that for large-volume BPH (≥80 mL), LEP may be associated with faster perioperative recovery, with ThuLEP associated with the shortest hospital stay. Given that the LEP group encompassed both HoLEP and ThuLEP, differences between these two laser modalities should be considered when interpreting pooled LEP outcomes. RASP may carry a lower risk of urinary incontinence, lower post-void residual volume, and more favorable long-term symptom control as assessed by IPSS. Both procedures demonstrated comparable safety, with no significant differences in low- or high-grade complications. Given the predominantly retrospective design and marked heterogeneity across studies, these pooled results should be interpreted with caution, and individualized surgical decision-making remains warranted. Further well-designed prospective randomized controlled trials with long-term follow-up are needed to validate these findings and support evidence-based clinical practice.

## Data Availability

The original contributions presented in the study are included in the article/Supplementary material, further inquiries can be directed to the corresponding authos.

## Footnotes

1
http://www.revman.cochrane.org
