# Supplementary material for: Robot-assisted simple prostatectomy vs. laser enucleation of the prostate for large-volume benign prostatic hyperplasia (BPH, ≥80 mL): a systematic review and meta-analysis
Source: Front Med (Lausanne). 2026 May 5;13:1804731. doi: 10.3389/fmed.2026.1804731 (PMC13183796; doi:10.3389/fmed.2026.1804731)
Supplement: Supplementary file 1 [file Supplementary_file_1.DOCX]

**Supplementary File 1**

**Table.1 Study characteristics**

| **Study** | **Country** | **Research** | **Study** | **Intervention** | **Patients** | **Age** | **Minvolume** | **IPSS** | **Quality** |
| --- | --- | --- | --- | --- | --- | --- | --- | --- | --- |
|  |  | **period(y)** | **design** |  | **(n)** | **(y)** | **/mass(ml/g)** |  | **score** |
| Chou et al.(14) | USA | 2018-2022 | Retrospective | RASP | 80 | 76.0±5.9 | ≥80g | 26.5±5.3 | 8 |
|  |  |  |  | HoLEP | 80 | 74.0±9.0 |  | 27.0±5.2 |  |
| Hartung et al.(15) | Germany | 2015-2021 | Retrospective | RASP | 39 | 70.6±7.9 | ≥80ml | 21.4±6.4 | 8 |
|  |  |  |  | HoLEP | 38 | 70.7±8.6 |  | 18.5±7.5 |  |
| Silvia et al.(16) | Spain | 2007-2023 | Retrospective | RASP | 50 | 72.4±7.8 | ≥150ml | 21.9±4.9 | 7 |
|  |  |  |  | HoLEP | 95 | 72.4±8.9 |  | 21.7±4.9 |  |
| Bove et al.(17) | Italy | 2011-2021 | Retrospective | RASP | 43 | 72.0±8.1 | ≥80g | 20.0±3.0 | 8 |
|  |  |  |  | HoLEP | 54 | 70.0±5.9 |  | 20.0±5.9 |  |
| Palacios et al.(18) | USA | 2019-2022 | Retrospective | RASP | 50 | 70.0±5.9 | ≥80ml | 23.0±9.6 | 8 |
|  |  |  |  | HoLEP | 90 | 72.0±5.2 |  | 18.0±9.6 |  |
| Vander et al.(19) | Belgium | 2009-2020 | Retrospective | RASP | 31 | 73.0±9.3 | ≥200ml | 18.0±9.6 | 8 |
|  |  |  |  | HoLEP | 22 | 77.5±7.4 |  | 15.0±5.2 |  |
| Kim et al.(20) | Korea | 2018-2021 | Retrospective | RASP | 33 | 68.1±10.1 | ≥80ml | NA | 7 |
|  |  |  |  | HoLEP | 26 | 70.8±7.6 |  |  |  |
| Fuschi et al.(21) | Italy | 2016-2018 | Prospective | RASP/LSP | 68 | 66.4±7.5 | ≥120mL | 23.9±1.9 | 8 |
|  |  |  |  | HoLEP | 42 | 68.2±6.1 |  | 24.2±3.0 |  |
| Umari et al.(22) | Italy | 2008-2015 | Retrospective | RASP | 81 | 69.0±7.4 | ≥100ml | 25.0±5.9 | 8 |
|  |  |  |  | HoLEP | 45 | 74.0±8.9 |  | 21.0±6.7 |  |
| Zhang et al.(23) | USA | 2008-2015 | Retrospective | RASP | 32 | 71.0±8.0 | ≥80g | 24.0±4.0 | 8 |
|  |  |  |  | HoLEP | 600 | 71.0±8.0 |  | 20.0±7.0 |  |
| Audige et al.(24) | France | 2020-2023 | Retrospective | RASP | 106 | 70.5±5.9 | ≥80ml | 20.9±6.2 | 8 |
|  |  |  |  | ThuLEP | 128 | 71.9±8.5 |  | 17.6±7.6 |  |
| Perri et al.(25) | Italy | NA | Retrospective | RASP | 100 | 70.2±8.3 | ≥150ml | 24.3±5.8 | 8 |
|  |  |  |  | ThuLEP | 100 | 74.5±7.6 |  | 22.5±7.6 |  |
| Hartung et al.(26) | Germany | 2015-2021 | Retrospective | RASP | 38 | 70.0±8.0 | ≥80ml | NA | 7 |
|  |  |  |  | ThuLEP | 38 | 70.0±6.0 |  |  |  |
| Susan et al.(27) | USA | 2017-2021 | Retrospective | RASP | 33 | 68.0±5.9 | ≥80g | 26.5±6.7 | 7 |
|  |  |  |  | ThuLEP | 69 | 68.0±8.1 |  | 22.0±12.6 |  |
| Hou et al.(28) | China | 2014-2020 | Retrospective | RASP | 15 | 66.4±6.4 | ≥80g | \| 26.3±5.1 \| \| --- \| | 7 |
|  |  |  |  | ThuLEP | 41 | 71.9±8.5 |  | 25.1±5.5 |  |

RASP,robot-assisted simple prostatectomy;HoLEP,holmium laser enucleation of the prostate;ThuLEP,thulium laser enucleation of the prostate;

IPSS,international prostate symptom score;NA,not available;

**Table.2 Baseline characteristics of patients.**

| Study | Intervention | BMI | PSA | PV | QoL | Qmax | Indwelling |
| --- | --- | --- | --- | --- | --- | --- | --- |
|  |  | (kg/m2) | (ng/mL) | (mL) |  | (mL/s) | catheter(n) |
| Chou et al.(14) | RASP | 28.5±5.2 | 5.2±2.5 | NA | NA | NA | 7 |
|  | HoLEP | 26.5±5.3 | 5.5±3.7 |  |  |  | 11 |
| Hartung et al.(15) | RASP | 29.0±5.9 | 10.1±6.1 | 186.7±55.7 | NA | NA | 27 |
|  | HoLEP | 30.2±6.2 | 12.3±10.8 | 184.8±54.9 |  |  | 13 |
| Silvia et al.(16) | RASP | NA | 7.7±4.2 | 203.4±98.1 | NA | 8.1±3.5 | 10 |
|  | HoLEP |  | 8.73±8 | 187.7±45.9 |  | 10.9±5.8 | 40 |
| Bove et al.(17) | RASP | 25.0±4.4 | 4.9±4.5 | 105.0±25.9 | 3.0±0.7 | 7.0±4.4 | NA |
|  | HoLEP | 26.0±3.0 | 5.1±3.3 | 102.0±20.7 | 3.0±0.7 | 7.0±2.2 |  |
| Palacios et al.(18) | RASP | 29.0±4.4 | 9.0±5.9 | 169.0±71.1 | 5.0±1.5 | 6.0±1.5 | 26 |
|  | HoLEP | 26.0±4.4 | 6.0±4.4 | 129.0±37.0 | 4.0±1.5 | 6.0±3.7 | 50 |
| Vander et al.(19) | RASP | 27.6±3.9 | 10.4±9.0 | 225.0±29.6 | 4.0±1.5 | 10.0±5.2 | 8 |
|  | HoLEP | 26.1±4.0 | 7.5 ±3.0 | 204.5±14.8 | 4.0±0.7 | 8.2±0.9 | 3 |
| Kim et al.(20) | RASP | NA | NA | 97.9±37.9 | NA | NA | NA |
|  | HoLEP |  |  | 84.1±31.3 | NA | NA | NA |
| Fuschi et al.(21) | RASP/LSP | 14.0±20.5 | 5.5±3.0 | 146.9±30.7 | 3.9±0.9 | 7.2±1.3 | 16 |
|  | HoLEP | 23.5±3.3 | 5.6±3.3 | 142.2±30.1 | 3.9±0.8 | 7.1±1.9 | 11 |
| Umari et al.(22) | RASP | 27.0±5.2 | 7.1±6.2 | 130.0±27.4 | NA | 8.0±4.4 | 9 |
|  | HoLEP | 26.0±3.7 | 8.6±8.4 | 130.0±58.5 |  | 9.0±5.2 | 4 |
| Zhang et al.(23) | RASP | NA | NA | NA | NA | NA | 11 |
|  | HoLEP |  |  |  |  |  | 140 |
| Audige et al.(24) | RASP | 26.5±2.9 | 10.9±7.2 | 135.2±39.7 | 4.4±0.7 | 8.9±3.0 | 30 |
|  | ThuLEP | 26.6±4.0 | 7.3±7.9 | 106.4±26.8 | 4.1±1.5 | 10.4±7.2 | 29 |
| Perri et al.(25) | RASP | NA | 4.9±2.3 | 186.3±12.3 | NA | 7.2±3.3 | 14 |
|  | ThuLEP |  | 4.2±1.7 | 178.6±11.4 |  | 9.0±2.8 | 12 |
| Hartung et al.(26) | RASP | 29.0±6.0 | NA | 184.0±51.0 | NA | NA | NA |
|  | ThuLEP | 29.0±4.0 |  | 179.0±44.0 |  |  |  |
| Susan et al.(27) | RASP | 28.2±5.0 | NA | 120.0±204.4 | NA | NA | 14 |
|  | ThuLEP | 28.1±4.0 |  | 180.0±334.8 |  |  | 32 |
| Hou et al.(28) | RASP | NA | 10.4±5.4 | 116.4±17.9 | 5.3±0.6 | 5.4±1.8 | 4 |
|  | ThuLEP |  | 8.7±7.5 | 89.8±7.8 | 5.1±0.7 | 6.7±4.1 | 17 |

BMI,body mass index;PSA,prostate-specific antigen;QoL,quality of life score;Qmax,maximum urinary flow rate;PVR,postvoid residual volume;PV:prostate volume;RASP,robot-assisted simple prostatectomy;HoLEP,holmium laser enucleation of the prostate;ThuLEP,thulium laser enucleation of the prostate;NA,not available;
