# Supplementary material for: Robot-assisted simple prostatectomy vs. laser enucleation of the prostate for large-volume benign prostatic hyperplasia (BPH, ≥80 mL): a systematic review and meta-analysis
Source: Front Med (Lausanne). 2026 May 5;13:1804731. doi: 10.3389/fmed.2026.1804731 (PMC13183796; doi:10.3389/fmed.2026.1804731)
Supplement: Supplementary file 2 [file Supplementary_file_2.DOCX]

**Supplementary File 2.**

Quality evaluation of the eligible studies with Newcastle–Ottawa scale.

*indicates criterion met; - indicates significant of criterion not met.
