# Supplementary material for: Robot-assisted simple prostatectomy vs. laser enucleation of the prostate for large-volume benign prostatic hyperplasia (BPH, ≥80 mL): a systematic review and meta-analysis
Source: Front Med (Lausanne). 2026 May 5;13:1804731. doi: 10.3389/fmed.2026.1804731 (PMC13183796; doi:10.3389/fmed.2026.1804731)
Supplement: Supplementary file 3 [file Supplementary_file_3.DOCX]

Supplementary File 3.

Sensitivity analysis of perioperative outcomes, complications and urinary incontinence.


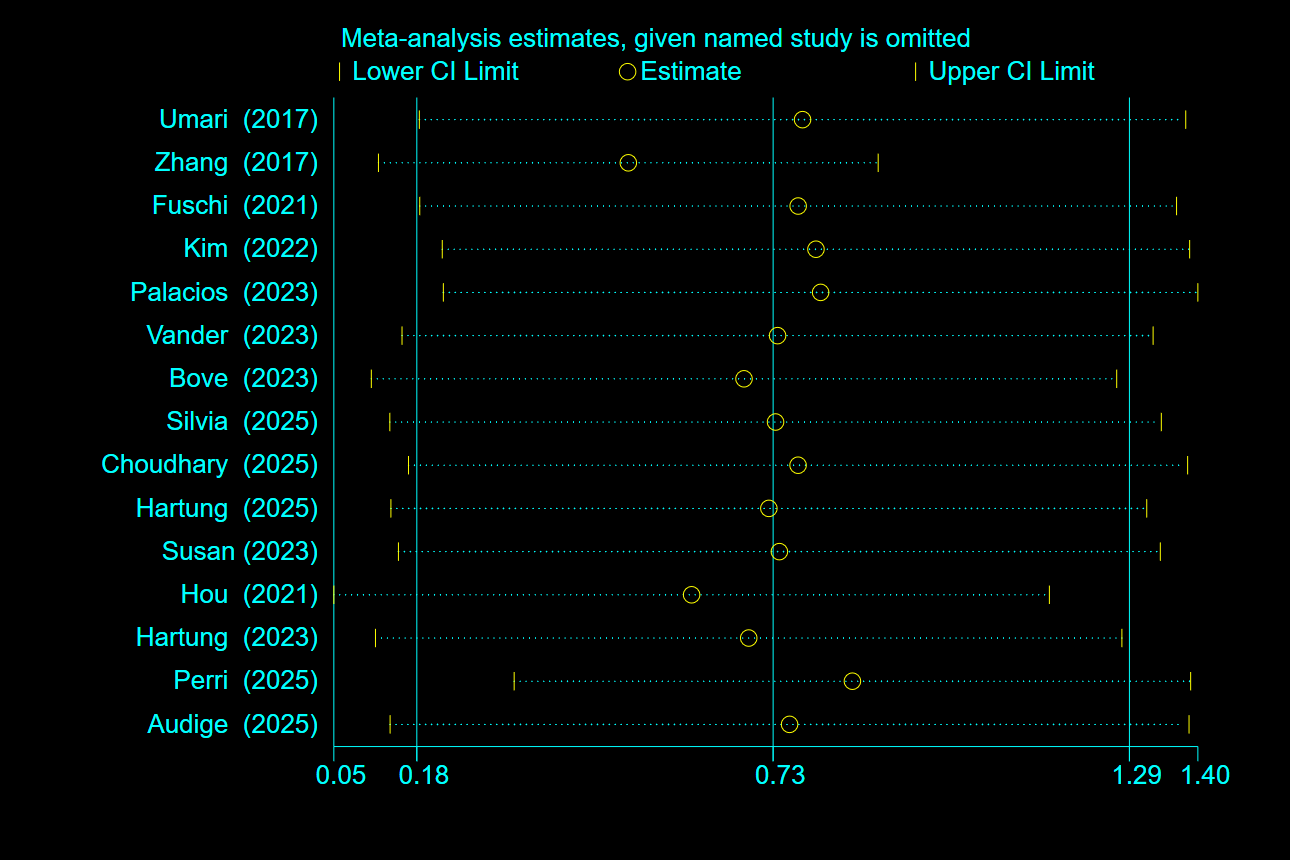


A:Sensitivity analysis of operative time by leave-one-out method.


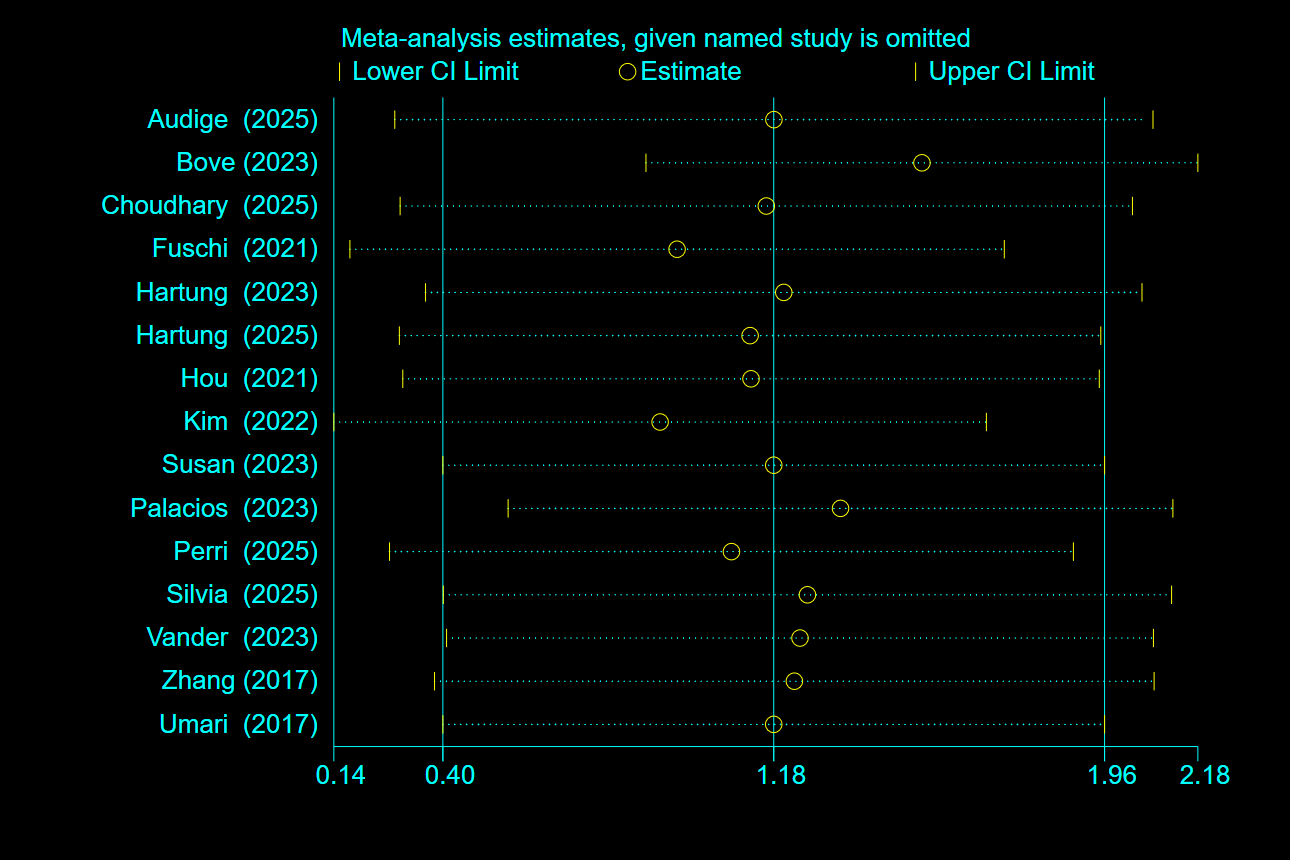


B:Sensitivity analysis of length of hospital stay by leave-one-out method.


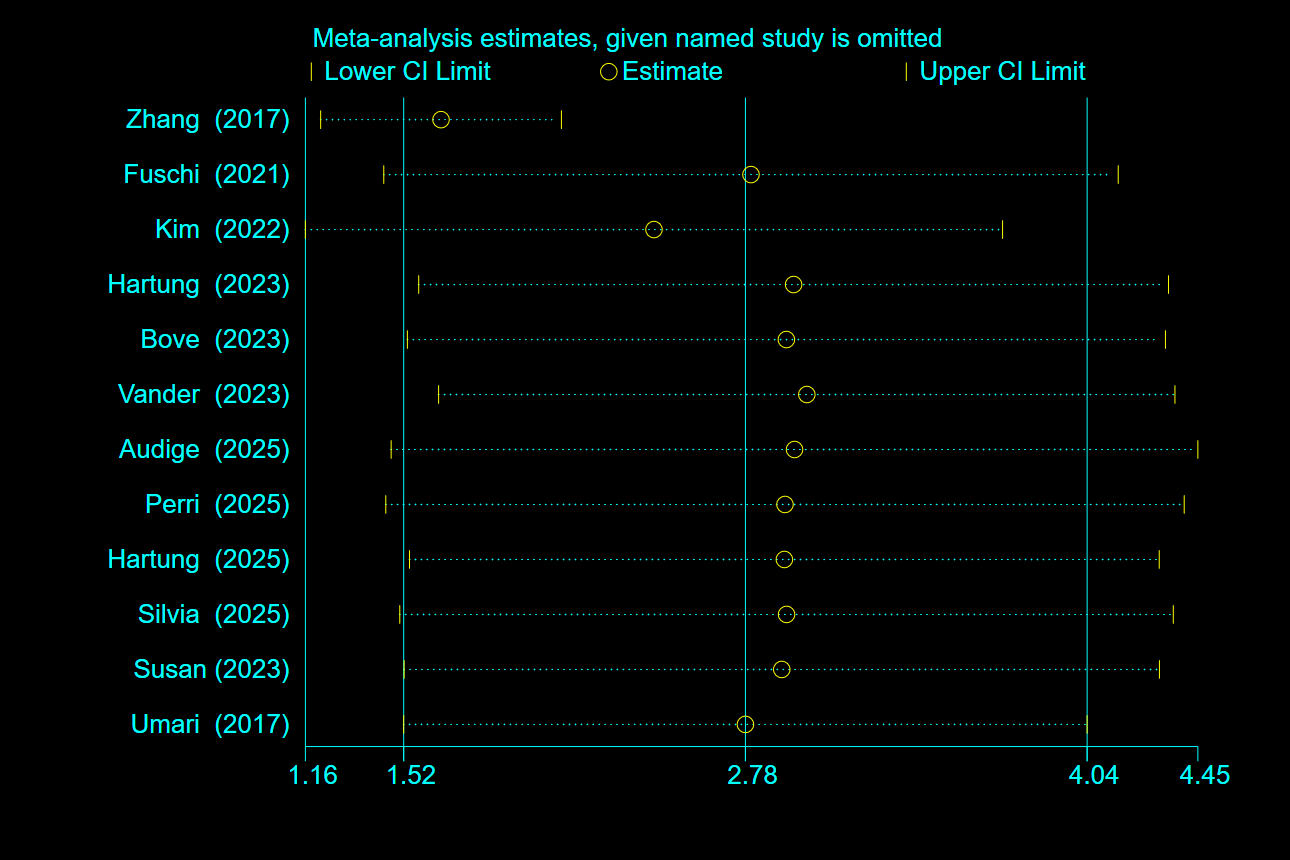


C: Sensitivity analysis of indwelling catheter time by leave-one-out method.

**
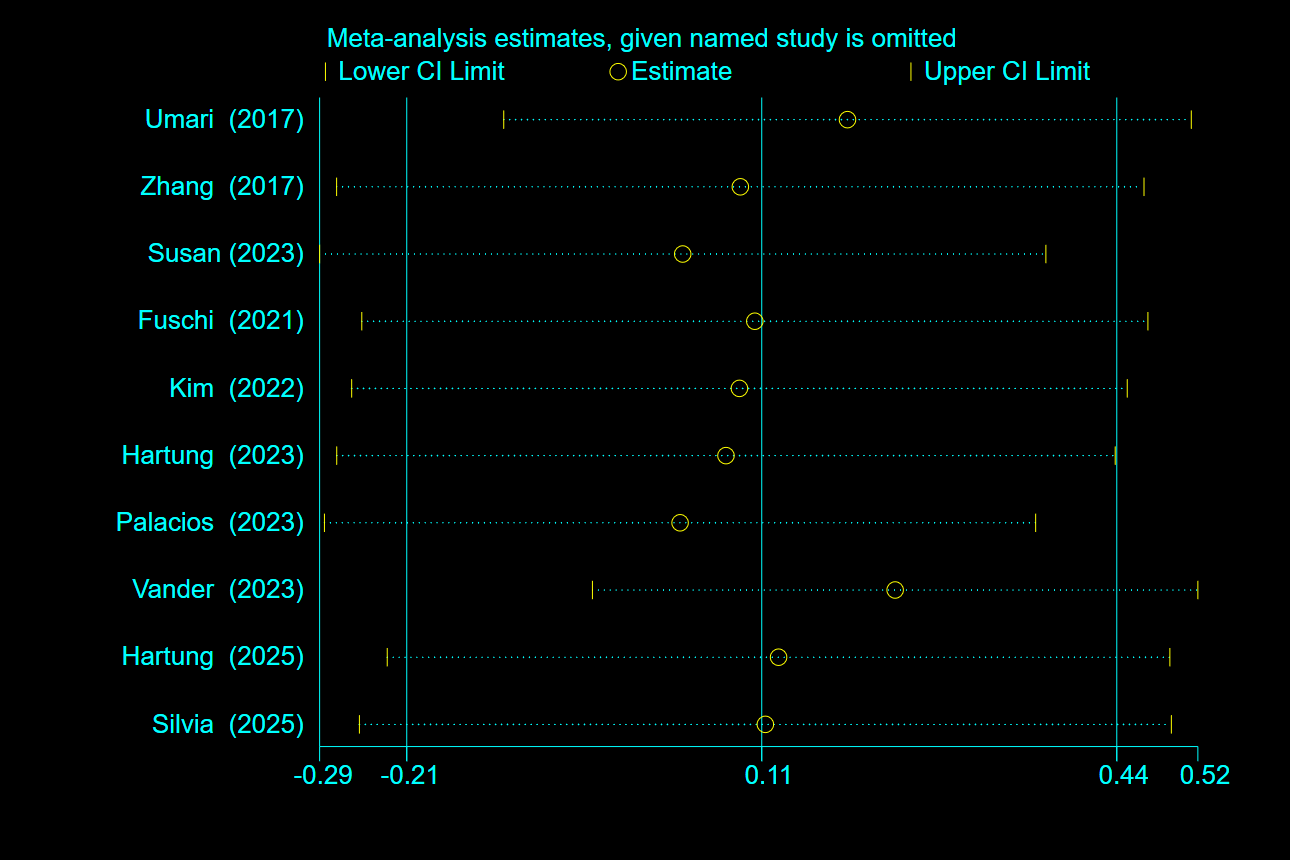
**

D: Sensitivity analysis of specimen weight by leave-one-out method.


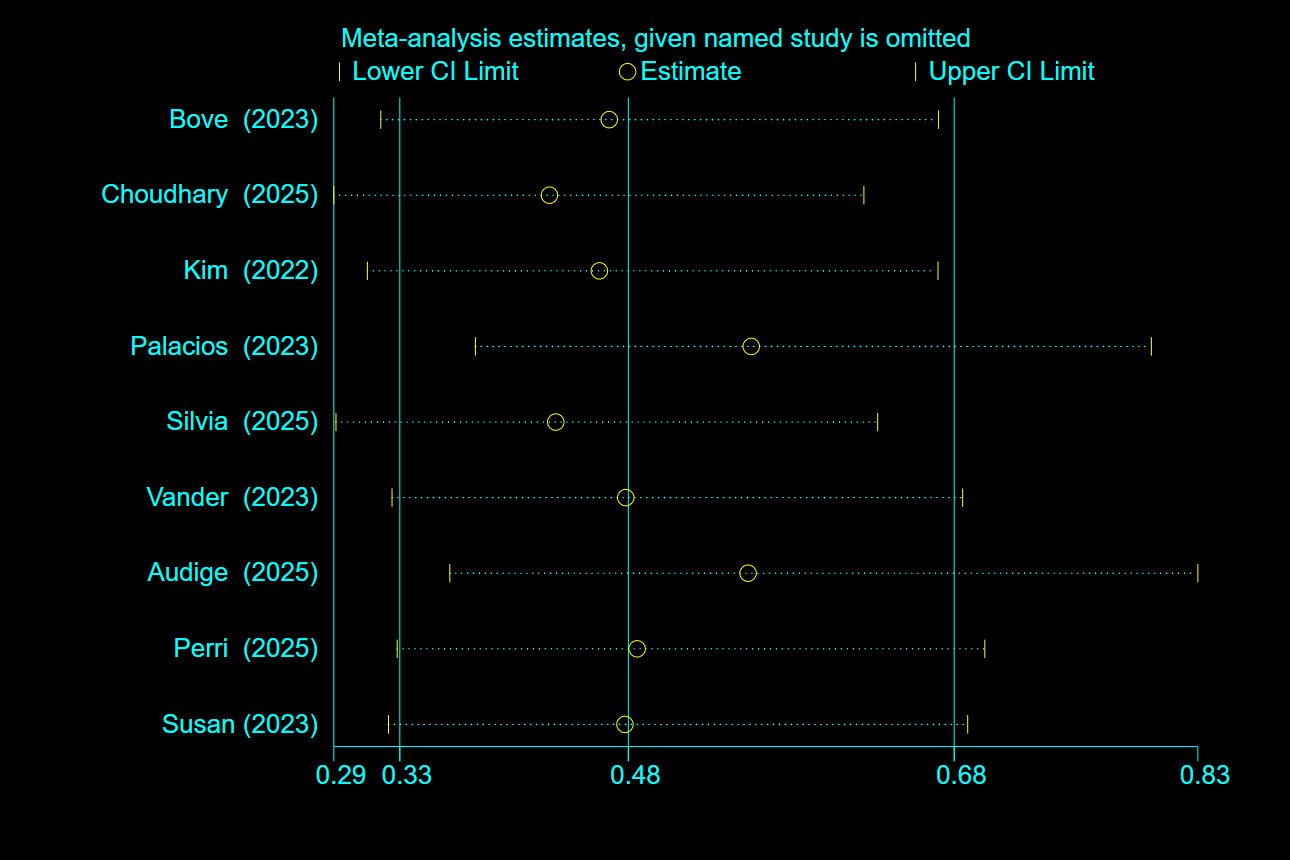


E: Sensitivity analysis of urinary incontinence by leave-one-out method.


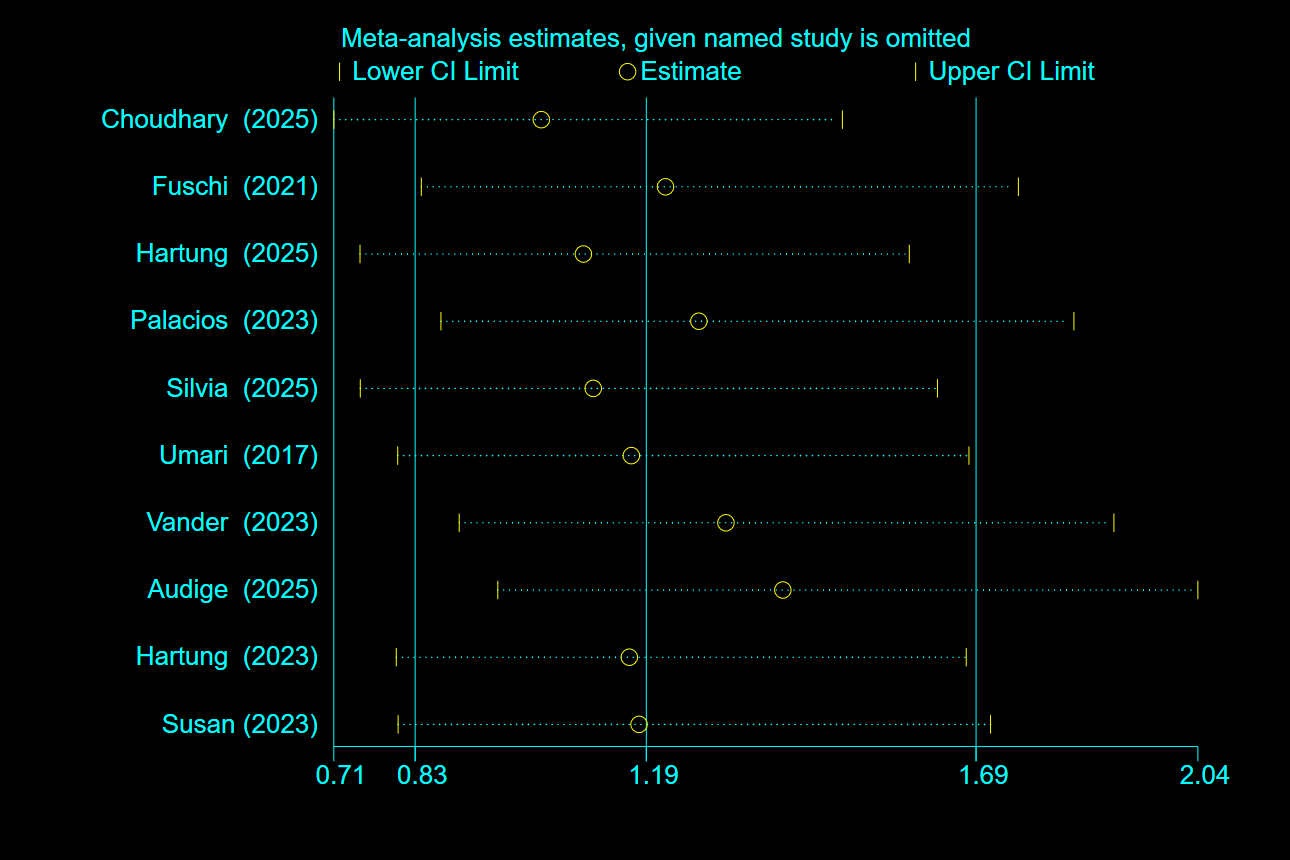


F: Sensitivity analysis of CDC ≤ II by leave-one-out method.


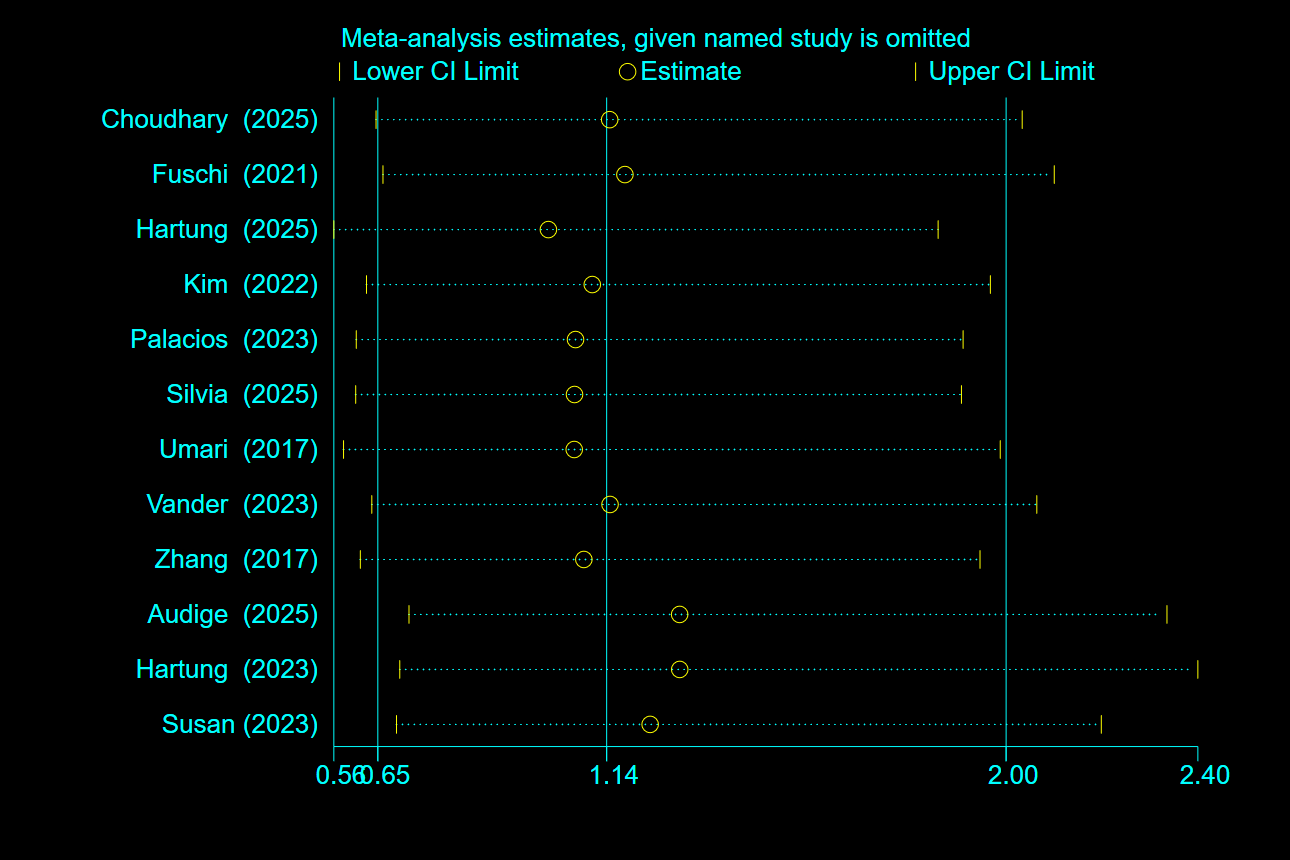


G: Sensitivity analysis of CDC > II by leave-one-out method
